# Development and Validation of an Indirect and Blocking ELISA for the Serological Diagnosis of African Swine Fever

**DOI:** 10.3390/pathogens13110981

**Published:** 2024-11-08

**Authors:** Chukwunonso Onyilagha, Kaye Quizon, Dmytro Zhmendak, Ian El Kanoa, Thang Truong, Thanuja Ambagala, Alfonso Clavijo, Van Phan Le, Shawn Babiuk, Aruna Ambagala

**Affiliations:** 1 National Centre for Foreign Animal Disease, Canadian Food Inspection Agency, Winnipeg, MB R3E 3M4, Canada; 2 National Microbiology Laboratory, Public Health Agency of Canada, Winnipeg, MB R3E 3M4, Canadathang.truong@phac-aspc.gc.ca (T.T.); 3 National Bio and Agro-Defense Facility, Agricultural Research Service, United States Department of Agriculture, Manhattan, KS 66506, USA; 4 Department of Microbiology and Infectious Disease, College of Veterinary Medicine, Vietnam National University of Agriculture, Hanoi 100000, Vietnam; 5 Department of Immunology, University of Manitoba, Winnipeg, MB R3E 0T5, Canada; 6 Department of Comparative Biology, Faculty of Veterinary Medicine, University of Calgary, Calgary, AB T2N 1N4, Canada

**Keywords:** African swine fever, diagnostics, indirect ELISA, blocking ELISA, serology

## Abstract

African swine fever (ASF) is an economically devastating viral disease of pigs caused by the ASF virus (ASFV). The rapid global spread of ASF has increased the demand for ASF diagnostics to be readily available and accessible. No commercial ASF enzyme-linked immunosorbent assay (ELISA) kits are manufactured and licensed in North America. Here, we report the development of two serological diagnostic assays, a blocking ELISA (bELISA) based on ASFV glycoprotein p54 and an indirect ELISA (iELISA) based on ASFV glycoproteins p54 and p72. The assays showed high sensitivity and specificity and detected anti-ASFV antibodies in serum samples from experimentally infected animals as early as 8 days post-infection. The two assays were produced commercially (AsurDx^™^ bELISA and iELISA) and subjected to extensive validation. Based on data from a set of characterized reference sera, the prototype commercial assays, while maintaining 100.00% specificity, showed 97.67% (AsurDx^™^ bELISA) and 83.72% (AsurDx^™^ iELISA) sensitivity. Both prototype assays detected anti-ASFV antibodies in serum samples collected from pigs experimentally infected with multiple ASFV strains and field samples collected from sick, recovering, and vaccinated animals. The two commercially available assays can be used in routine ASF diagnostics, serological surveys, and for evaluating serological responses to ASF vaccine candidates.

## 1. Introduction

African swine fever (ASF) is a fatal hemorrhagic disease of swine. The causative agent, ASF virus (ASFV), is a complex double-stranded DNA (dsDNA) virus with a genome ranging from 170 to 194 kbp [1,2] encoding 150–167 proteins. ASFV is the sole member of the *Asfarviridae* family and shares some features such as its genome structure and cytoplasmic replication with other large dsDNA viruses such as Poxviruses and Iridoviruses [3]. Depending on the virulence of the ASFV strain, clinical signs in infected pigs can vary from mild fever and a lack of appetite to high fever and 100% mortality [4]. Highly virulent ASFV strains cause acute infections characterized by fever, hemorrhages, depression, and ataxia, ultimately leading to the death of infected animals between 4 and 15 days post-infection (dpi) [2]. Moderately virulent strains of ASFV show similar but less marked clinical signs, and the mortality rate of affected pigs can vary from 30% to 70%. Low-virulent ASFV strains lead to chronic ASF characterized by intermittent fever, multifocal necrosis in the skin, and arthritis [5].

ASF-affected countries mainly rely on early detection, quarantine, slaughter, and disinfection to control the disease. The early detection of ASF is routinely performed using highly sensitive and specific molecular assays to detect ASFV genomic material. Serological detection is critical for regaining disease-free status following an acute outbreak and for the detection of pigs infected with low-virulent strains. In ASF endemic countries, many naturally attenuated and genetically modified attenuated ASFV strains have been detected recently [6,7,8,9,10,11,12]. These attenuated ASFV strains cause no or mild clinical signs and can go unnoticed, leading to widespread disease. The lack of or presence of merely mild clinical signs and low intermittent viremia observed in pigs infected with attenuated ASFV strains make it more difficult to detect these viruses using molecular assays, but they will most likely be detected during serological surveillance. Antibodies against ASFV appear early (~7–10 dpi) and persist for a long period, sometimes for many years [13]. Therefore, the presence of anti-ASFV antibodies could be a sign of an ongoing or previous ASFV infection. Although there are no globally accepted vaccines for ASF, two live-attenuated recombinant vaccines are licensed in Vietnam [14]. Antibodies against ASFV can be used to measure the immune responses to these vaccines.

ASFV-infected animals induce strong antibody responses to the highly conserved ASFV structural proteins p30, p54, and p72; therefore, they are ideal antigens for developing serological assays [15,16,17,18]. Several enzyme-linked immunosorbent assays (ELISAs) for detecting anti-ASFV antibodies have been developed and commercialized. However, none of the kits are manufactured in North America; therefore, they may not be easily accessible in the event of an outbreak. In addition, there is a need to develop more sensitive ELISA kits to address the issue of low sensitivity associated with some of the commercially available kits.

This study describes the development and extensive validation of highly sensitive and specific bELISA, based on ASFV p54, and iELISA, based on ASFV p54 and p72, for detecting anti-ASFV antibodies in porcine serum samples, which can be licensed in North America.

## 2. Materials and Methods

### 2.1. Recombinant Antigen Production

Three well-known highly immunogenic ASFV proteins, p30, p54, and p72, were selected to develop a highly sensitive and specific ELISA for ASFV. The full-length gene sequences of the targeted proteins were extracted from the ASFV whole genome sequence of Georgia 2007/1 isolate (GenBank Accession #FR682468.1). The sequences were codon-optimized for optimal expression in insect cells, synthesized, and cloned into a pAB-bee™ –FH vector containing a polyhedron promoter (AB Vector LLC, San Diego, CA, USA) by GenScript (GenScript USA Inc, Piscataway, NJ, USA). The purified pAB-bee™–FH vector containing the ASFV p30, p54, and p72 genes was co-transfected with linearized baculovirus vector DNA, Pro-Fold™-ER1 (AB Vector LLC, San Diego, CA, USA), into Sf9 insect cells (Expression Systems LLC, Davis, CA, USA) to generate a recombinant baculovirus containing p30, p54, and p74 genes. These recombinant baculoviruses were plaque-purified, and the selected clones were sub-cultured at 27 °C in Sf9 cells with shaking at 130 RPM for 120 h. For protein expression, the sub-cultures were inoculated onto suspension cultures of Trichoplusia ni (Tni) cells (Expression Systems LLC, Davis, CA, USA) at a multiplicity of infection (MOI) of 5 to 10. The infected cells were harvested 72 h post-infection and pelleted by centrifugation at 1500 RCF for 20 min at 4 °C. The 8xHis-tagged proteins were purified from the cell pellets using Ni-NTA agarose beads (Qiagen, Toronto, ON, Canada).

### 2.2. Western Blotting

Tni culture pellets were resuspended in an equivalent amount of Dulbecco’s phosphate-buffered saline (Wisent Bioproducts, St-Bruno, QC, Canada), and 65 µL of the suspension was mixed with 25 µL of the sample buffer (Invitrogen, Waltham, MA, USA) and 10 µL of 1M DTT (Thermo Fisher Scientific, Waltham, MA, USA) and heated at 70 °C for 10 min. The reduced samples were then loaded into pre-cast polyacrylamide gels (Invitrogen, Waltham, MA, USA), and the proteins were separated by electrophoresis in MOPS running buffer (Invitrogen, Waltham, MA, USA) at 100 V for one hour. Immediately after electrophoresis, the separated proteins were transferred to a PVDF membrane (Invitrogen, Waltham, MA, USA) for 7 min using the iBlot^®^ Gel Transfer Device (Invitrogen, Waltham, MA, USA). The PVDF membrane was blocked using 1× blocking buffer (Sigma-Aldrich, Oakville, ON, Canada) at room temperature for one hour, and polyclonal anti-ASFV serum from an experimentally infected pig was applied at 1/20 in 1× blocking buffer at room temperature for one hour. After washing, horseradish peroxidase (HRP)-conjugated rabbit anti-swine IgG (Sigma-Aldrich, Oakville, ON, Canada) was applied at 1/2000 at room temperature for one hour. The bands were visualized using a chemiluminescent reagent (Azure Biosystems, Dublin, CA, USA). The slight shifts in the expected molecular weight of the proteins could be explained by the presence of his-tag, which was not cleaved after the protein expression.

### 2.3. Serum and Meat Exudate Samples

For the initial screening of ASFV antigens, a collection of known ASF antibody-negative porcine sera (*n* = 458) collected from pig farms across Canada (a historically ASF-free country) and 151 serum samples collected from pigs (serial bleed) experimentally inoculated with ASFV ASF OURT88/3 followed by ASFV Malta’78 was used.

The performance of the AsurDx^™^ ELISA kits (BioStone™ Animal Health LLC, Southlake, TX, USA) was further evaluated using archived serial bleed serum samples collected from pigs (four- to five-week-old Landrace-Large White cross-bred) experimentally inoculated with different ASFV strains at the NCFAD. The viruses include ASFV Estonia 2014 (p72 genotype II, moderately virulent) [11], ASFV Malta’78 (p72 genotype I, moderately virulent) [19], ASF Georgia 2007/1 (p72 genotype II, highly virulent) [20], ASFV OURT88/3 (p72 genotype I, low-virulent) [21,22], and ASFV-GUS-Vietnam (p72 genotype II, live-attenuated) [23]; they were propagated in primary porcine peripheral leukocyte culture (PPL) and titrated on primary porcine alveolar macrophages (PAM), as described previously [24].

To assess the sensitivity and specificity of the prototype AsurDx^™^ ELISA kits (BioStone™ Animal Health LLC, Southlake, TX, USA), 61 pre-characterized reference sera saved from the annual proficiency panels from the EU Reference Laboratory (EURL) for ASF (Madrid, Spain) were used; the serostatus of each sample was determined by EURL using the screening and confirmatory serological assays [ELISA, immunoblotting (IB), and indirect immunoperoxidase test (IPT)] for ASF.

The prototype commercial AsurDx^™^ iELISA and bELISA kits (BioStone™ Animal Health LLC, Southlake, TX, USA) were also evaluated using 4085 field samples comprising 2792 ASF-negative serum samples from Canada and the USA (both ASF-free countries) and 1293 sera obtained from pig farms in Vietnam (ASF endemic country). The samples from Canada and the US include 2406 serum samples from healthy commercial herds (Canada) and 186 and 200 samples from Canadian and US feral swine, respectively. The sera from Vietnam include 210 known ASF-negative pig sera from high bio-security commercial farms, 414 sera from ASF-recovering pigs, 310 sera from ASFV-infected pigs, and 359 sera from pigs vaccinated with a single dose of the AVAC ASF LIVE vaccine (AVAC Vietnam Joint Stock Company, Hung Yen, Vietnam). The recovering pigs are defined as those who survived a confirmed ASF outbreak on a farm; the sick pigs are those that displayed clinical signs consistent with ASF at the time of sample collection and tested positive for ASFV genomic material by real-time PCR.

To assess the suitability of the AsurDx^™^ ELISA kits (BioStone™ Animal Health LLC, Southlake, TX, USA) for testing meat exudate samples, archived meat exudate (diaphragm) samples from ASFV Malta’78-infected pigs were used.

### 2.4. Selection of ASFV Antigens for ELISA Development

Indirect ELISA was used to select the best antigens, and checkerboard titration was used to determine the optimal concentration of each antigen. Once optimum antigen concentrations were determined (i.e., 50 ng (p54) and 100 ng (p30 and p72)/well in 100 µL of carbonate-bicarbonate buffer at pH 9.6), the plates were coated with each antigen overnight at 4 °C. The next day, the plates were washed five times with PBS containing 0.05% Tween-20 (PBS-T), blocked with 100 µL/well 1× blocking buffer (Sigma-Aldrich, Oakville, ON, Canada) at 37 °C on an orbital shaker (600 RPM) for 60 min, and washed with PBS-T. The serum samples were diluted 1/20 in 1× blocking buffer and applied at 100 µL/well. The plates were incubated at 37 °C on an orbital shaker (600 RPM) for 60 min and washed five times with PBS-T, and horseradish peroxidase (HRP)-conjugated rabbit anti-swine IgG (Sigma-Aldrich) diluted 1/5000 in 1× blocking buffer was added to each well at 100 µL/well. After 60 min of incubation at 37 °C on an orbital shaker (600 RPM), the plates were washed five times, as described above; 3,3′,5,5′-Tetramethylbenzidine (TMB) ELISA substrate (KPL, Milford, MA, USA) was applied at 100 µL/well and incubated in darkness at room temperature for 10 min. The reaction was stopped using 1M H_2_SO_4_ (100 µL/well), and absorbance was measured at 450 nm within 10 min. All samples were tested in duplicate. Percent responses were calculated based on the anti-ASFV positive and negative serum controls used. The positive and negative controls originated from a single lot and were used on every test plate. The responses for iELISA were calculated as sample percent positivity as follows: Percent positivity = (sample OD at A450 nm ÷ positive control OD at A450 nm) × 100.

### 2.5. Monoclonal Antibody Production Against ASFV p54

Four BALB/c mice were inoculated subcutaneously, each with 30 µg of purified recombinant baculovirus ASFV p54 in 20% *v*/*v* Emulsigen^®^-D (MVP adjuvants, Omaha, NE, USA) in a total volume of 100 µL, eight times and 28 days apart. A final immunization of 5 µg of ASFV p54 in PBS (100 µL) was administered intravenously, and three days later, the mice were euthanized; splenocytes were harvested and fused with a mouse myeloma cells line (ATCC CRL-1581, Manassas, VA, USA) [17]. The resulting hybridomas were then repeatedly subcloned and screened for p54-specific antibodies by indirect ELISA until clonal populations were achieved. Thirty-seven unique monoclonal antibodies that recognize ASFV p54 in iELISA were generated, of which 16 were able to compete with porcine serum containing anti-ASFV antibodies. The monoclonal antibody, F131-G12, which showed the highest competition, was selected to develop a blocking ELISA.

### 2.6. Indirect Immunoperoxidase Test (IPT)

The EURL for the ASF (EURL-ASF)-developed IPT assay [25] was used as the confirmatory assay for the presence of ASFV-specific antibodies in experimental samples and in a selected number of discrepant samples. The standard operating procedure provided by the EURL-ASF [26] was followed to complete the assay.

### 2.7. AsurDx^™^ Blocking ELISA (AsurDx^™^ bELISA)

Using the F131-G12 ASFV p54 monoclonal antibody, in collaboration with BioStone™ Animal Health LLC (Southlake, TX, USA), a prototype AsurDx^™^ ASF antibody bELISA was developed. The assay was optimized and carried out as described below. The ASFV p54-coated plate (stored at 4 °C) was brought to room temperature, and the positive and negative controls were applied to the plate at 100 µL/well. Samples were added at 100 μL/well (1/2 dilution in assay diluent) and mixed by gently rocking the plate for 1 min. The plate was covered with foil and incubated at 37 °C for 30 min. The plate was washed five times by adding 250 μL of 1× wash solution to each well. The streptavidin-HRP conjugate (50 μL/well) and Anti-ASF mAb-Biotin (50 μL/well) were added to the plate, and the solution was mixed by gently rocking the plate manually for 1 min. The plate was covered with foil and incubated at 37 °C for 30 min, washed five additional times, and tapped dry on a paper towel before 100 μL of the TMB substrate was added. After 15 min of incubation at room temperature, 100 μL of the stop solution was added to each well, and the optical density (OD) of each well was read at 450 nm. The inhibition percentage (IP) was calculated as follows: IP (%) = [1 − (OD450 test sample/Mean OD450 Negative Control)] × 100. Result interpretation: IP ≥ 50%, positive; IP < 40%, negative; 50% > IP ≥ 40%, suspicious.

### 2.8. AsurDx^™^ Indirect ELISA (AsurDx^™^ iELISA)

The prototype AsurDx^™^ ASF antibody iELISA test kit (developed at BioStone™ Animal Health LLC, Southlake, TX, USA), which is based on a mixture of recombinant ASFV proteins, p54 and p72, was carried out according to the manufacturer’s instructions. Briefly, the ASF p54/p72 antigen-coated plate and all reagents were brought to room temperature for at least an hour. The positive and negative controls were added at 50 μL/well, and the previously diluted sample (1/40) was added at 100 μL/well. The plate was covered with foil and incubated at 37 °C for 30 min before being washed (five times) by adding 250 μL of 1× wash buffer solution to each well. After the washing step, 100 μL of 1× HRP-conjugated antibody solution was added to each well of the plate. The plate was covered with foil, incubated for 30 min at 37 °C, and washed three times before 100 μL of the TMB substrate was added to each well. The plate was covered and kept at room temperature for 15 min before adding 100 μL of the stop solution. The OD of each well was read at 450 nm, and the percent positivity (PP) was calculated as follows: PP (%) = (OD450 test sample/mean OD450 positive control) × 100. Result interpretation: PP ≥ 25%, positive; PP < 20%, negative; 20% ≥ PP > 25%, suspicious.

### 2.9. INgezim PPA COMPAC Blocking ELISA (PPA)

The commercially available INgezim PPA COMPAC^®^ blocking ELISA (Gold Standard Diagnostics, Madrid, Spain) was used to test sera according to the manufacturer’s instructions. Briefly, the pre-coated PPA plate was allowed to equilibrate to room temperature for at least 30 min. Then, 100 µL of a 1/2 dilution of controls or sera using the included diluent was applied to the plate in duplicate. The plate was incubated for one hour at 37 °C. The samples were removed, and the plate was washed four times using the included washing solution. The peroxidase-labelled monoclonal antibody (mAb) specific to ASFV p72 was diluted 1/100 and applied at 100 µL per well. The plate was incubated for 30 min at 37 °C. The plate was washed five times. The TMB substrate was applied at 100 µL and allowed to develop for 15 min in darkness. The stop solution was applied at the end of the incubation time, and the plate was read at 450 nm within 5 min of completion. The blocking percentage was calculated as follows: Blocking % (x%) of a sample = ((NC − Sample OD)/(NC − PC)) × 100. Result interpretation: x% ≥ 50%, positive; x% < 40%, negative; 50%> x% ≥ 40%, suspicious.

### 2.10. ID. Screen^®^ ASF Indirect ELISA (ID. Screen)

The commercially available ID. Screen^®^ ASF Indirect ELISA kit (Innovative Diagnostics, Grabels, France) is a multi-antigen indirect ELISA kit for detecting antibodies against ASFV p32, p62, and p72. This assay was used to test the experimental samples at the NCFAD together with other assays. The manufacturer’s recommended testing protocol was followed. All reagents were allowed to come to room temperature before performing the assay. Pre-diluted controls and samples (1/20) were added at 200 μL to each well as required and incubated for 45 min at 21 °C (±5 °C). The plate was washed three times with 300 µL/well of the wash solution. Then, 100 µL/well of the pre-diluted (1/10) conjugate was applied and incubated for 30 min at 21 °C (±5 °C). The plate was washed as described above, and 100 µL/well of the substrate solution was added to each well. The plate was incubated in the dark for 15 min at 21 °C (±5 °C) before applying the stop solution (100 μL/well). The OD was read at 450 nm. The ID. Screen S/P percentage (S/P%) was calculated using the following formula: S/P % = (Net Sample OD/Net PC OD) × 100. Result interpretation: S/P ≥ 40%, positive; S/P ≤ 30%, negative; 30% < S/P < 40%, suspicious. This assay was used to test the serum and meat exudate (ASFV Malta’78 only) samples collected from pigs experimentally infected with ASFV Estonia 2014, ASFV Malta’78, ASFV Georgia 2007/1, ASFV OURT88/3, and ASFV-GUS-Vietnam.

### 2.11. Real-Time PCR for the Detection of ASFV Genomic Material in Serum Samples

The MagMAX™ Pathogen RNA/DNA Kit and the MagMAX Express-96 Magnetic Particle Processor (Life Technologies, Burlington, ON, Canada) were used to perform total nucleic acid extractions from the serum samples following the manufacturer’s recommended protocol.

A TaqMan qPCR assay, which specifically amplifies a conserved region of the p72 gene of ASFV, was used to quantify the ASFV genomic material [27]. The TaqMan™ Fast Virus 1-Step master mix (Life Technologies, Burlington, ON, Canada) was used to perform the real-time PCR on Bio-Rad CFX96 Touch™ (Bio-Rad, Hercules, CA, USA) using the recommended cycling conditions (50 °C for 5 min; 95 °C for 20 s; followed by 40 cycles of 95 °C for 3 s and 60 °C for 30 s). Samples with cycle threshold (ct) values of 35.44 and lower were considered positive, values from 35.45 to 39.99 were deemed suspicious, and values over 39.99 were considered negative. As an internal control of optimal nucleic acid extraction, with the absence of PCR inhibitors in the samples, a TaqMan qPCR assay for beta-actin [28] was used.

### 2.12. Data Analysis

The receiver operating characteristic (ROC) curve analysis [29] package in R [30] was used to select the in-house cut-off threshold for the iELISA using IPT as a reference. The figures for the AsurDx^™^ bELISA and iELISA kits were graphically represented using GraphPad Prism version 9.3.1 for Windows (GraphPad Software, San Diego, CA, USA).

## 3. Results

### 3.1. Expression and Purification of ASFV p30, p54, and p72

Recombinant ASFV p30, p54, and p72 were expressed in baculovirus with a 6x-histidine (His) tag for purification. Protein sizes were confirmed (Figure S1) by Coomassie-stained gel (A) and Western blot (B) using anti-ASFV positive swine polyclonal serum. All three ASFV proteins were successfully expressed and purified, and their expected sizes (23 kDa, 20 kDa, and 73 kDa for p30, p54, and p72, respectively) were correctly observed when compared to a mass standard (Figure S1).

### 3.2. Selection of ASFV Antigens for the Development of ELISA

The purified ASFV antigens, p30, p54, and p72, were screened for their ability to be recognized by anti-ASFV antibodies in serial bleed sera and final bleeds (hyperimmune sera) from pigs repeatedly infected with ASFV OURT88/3 and subsequently challenged with ASFV Malta’78. The serostatus of each sample was confirmed using IPT before the screening.

**Figure 1:**
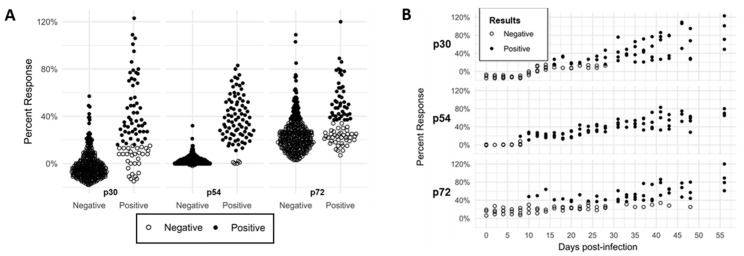
Direct comparison of in-house p30, p54, and p72 iELISA using paired serial bleed samples. Known-negative sera and serial bleed sera were collected from pigs infected repeatedly with ASFV OURT88/3 and subsequently challenged with ASFV Malta’78 and tested with the three in-house iELISA. The sample distribution (**A**) and the performance between the three in-house iELISA (**B**) were assessed. These data were used to select the p54 iELISA for further development. Open circle, negative; closed circle, positive.

The iELISA results for p54 showed a narrow distribution (Figure 1A), contributing to a lower cut-off threshold (10.5%) compared to that of p30 (15.5%) and p72 (36.5%), as calculated by ROC analysis (Table S1). Based on the 10.5% cut-off, the p54 iELISA demonstrated better sensitivity (88.4%) and specificity (94.8%) than the p72 (51.8% sensitive and 83.0% specific) and p30 (83.5% sensitive and 83.3% specific) assays. When the serial bleed serum samples were tested, the p54 iELISA detected anti-ASFV antibodies as early as 8 dpi (Figure 1B), two days before the p72 iELISA. The baculovirus-expressed p30 iELISA could not detect ASFV-specific antibodies in these samples until 16 dpi (Figure 1B). Collectively, of all three baculovirus-expressed ASFV antigens we evaluated, p54 performed the best; therefore, it was selected for developing a competitive/blocking ELISA. Blocking/competitive ELISA is unsuitable for detecting antibodies in some sample types, such as meat exudate, processing fluid, etc. Therefore, we also pursued developing an iELISA based on ASFV p54 and p72 antigens.

### 3.3. Performance of AsurDx^™^ bELISA and iELISA Using a Panel of Reference Sera and Known-Negative Field Sera from Canada, USA, and Vietnam

Following the successful optimization of the in-house ELISA, prototype versions of the assays, AsurDx^™^ iELISA and AsurDx^™^ bELISA, were manufactured in collaboration with BioStone™ Animal Health LLC. The AsurDx^™^ iELISA was manufactured based on p54 and p72, while the AsurDx^™^ bELISA was manufactured based on p54 and the monoclonal antibody F131-G12. To assess the sensitivity and specificity of the AsurDx^™^ ELISA kits, a panel of 61 characterized reference sera of known serostatus was used. Sensitivity is the ability of a test to score a positive sample as positive, and specificity is the ability of a test to score a negative sample as negative [31]. Based on data from 43 weak to strong positive reference sera, AsurDx^™^ kits showed a sensitivity of 97.67% (bELISA) and 83.72% (iELISA), while PPA showed 74.42% (Table 1). To determine the assay specificity based on 18 negative reference sera, the AsurDx^™^ and PPA kits all showed a specificity of 100.00% (Table 1).

**Figure 2:**
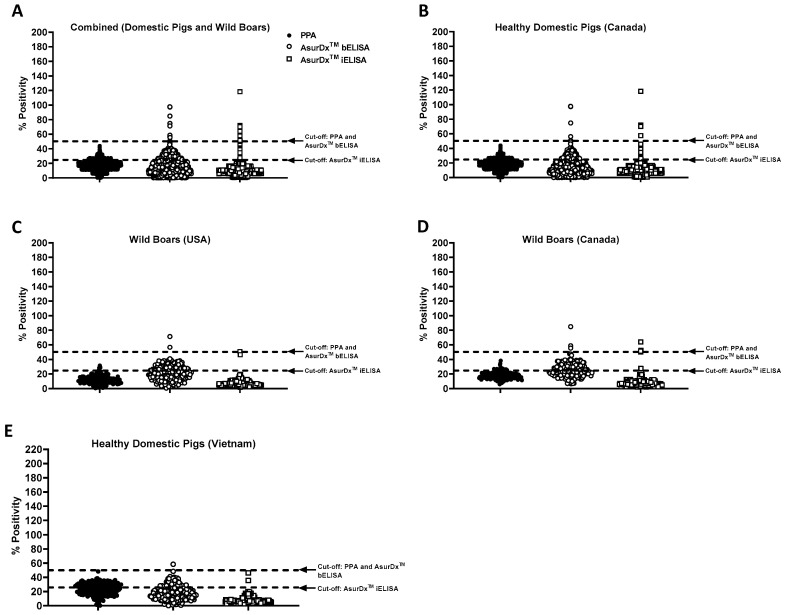
Performance of AsurDx^™^ ELISA kits with ASF-negative samples from North America and Vietnam. The performance of the AsurDx^™^ ELISA kits was assessed and compared to that of PPA using ASF-negative samples from North America (**A**), including healthy pigs from Canada (**B**), wild boars from the USA (**C**), and wild boars from Canada (**D**). The ASF-negative samples from healthy domestic pigs in Vietnam are shown in (**E**). Figure (**A**) is a summary of Figures (**B**–**D**). The cut-off for each assay is shown with a broken line.

In a different assay performance assessment, 2792 samples from North America (healthy pigs and wild boars from Canada and the USA) were tested, and the results were compared to those of PPA. There were no differences between the assay performances, as the AsurDx^™^ bELISA recorded 99.32% specificity while the AsurDx^™^ iELISA and PPA showed 99.36% and 99.93% specificity, respectively (Figure 2A). A few false-positives and suspicious results were observed in all three ELISA kits, which could be explained by the fact that those samples were mainly hemolyzed. To reduce the possibility of obtaining suspicious or false-positive results with the AsurDx^™^ ELISA kits, hemolyzed serum samples should not be used. A further breakdown of the assay performance by country or sample origin showed that AsurDx^™^ ELISA kits performed as well as PPA, with specificity ranging from 96.77% to 100% (Figure 2B,D and Table 2). To further evaluate the performance of the AsurDx^™^ ELISA kits using known-negative field samples from farms in Vietnam, the assays showed a specificity of 99.05% (AsurDx^™^ iELISA), 98.57% (AsurDx^™^ bELISA), and 99.52% (PPA) (Figure 2E and Table 2). Overall, these results show that the prototype AsurDx^™^ ELISA kits and the widely used PPA kit have comparable levels of specificity to ASFV, with the AsurDx^™^ bELISA having the potential to detect anti-ASFV antibodies in samples very early in the infection.

### 3.4. Performance of AsurDx™ bELISA and iELISA Using Samples from Pigs Experimentally Infected with Multiple ASFV Strains

The pigs were experimentally infected with different strains of ASFV, and the serial bleed serum samples were collected at different time points and assessed; the sera from these pigs were used to determine the sensitivity of the AsurDx^™^ ELISA kits compared to that of two commercially available ELISA kits (PPA and ID. Screen). The results obtained with ASFV Estonia 2014 showed that the AsurDx^™^ ELISA kits were more sensitive and detected anti-ASFV antibodies earlier than PPA and ID. Screen (Table 3 and Figure S2A). Similarly, the AsurDx^™^ ELISA kits (except AsurDx^™^ iELISA in ASF Malta’78) detected anti-ASFV antibodies earlier than PPA and ID. Screen in samples from ASFV Malta’78 (Table 3 and Figure S2B)-, ASFV Georgia 2007/1 (Table 3 and Figure S2C)-, and ASFV OURT88/3 (Table 3 and Figure S2D)-infected pigs. All the kits assessed performed in a similar manner with the samples obtained from the pigs infected with ASFV-GUS-Vietnam, detecting anti-ASFV antibodies around 9 dpi (Table 3 and Figure S2E). These results show that, in addition to the specificity of the AsurDx^™^ ELISA kits, they are relatively more sensitive than the other assessed commercially available ELISA kits when tested with most experimental sample sets.

### 3.5. Performance of AsurDx^™^ bELISA and iELISA Using Field Samples from ASFV-Infected Pigs in Vietnam

Because field samples play key roles in determining the effectiveness of any assay despite their complex nature, we wanted to further assess the performance of the AsurDx^™^ ELISA kits with samples obtained from the field. We obtained samples from Vietnam comprising pigs recovering from ASF (*n* = 412–414), pigs showing ASF clinical signs (sick pigs, *n* = 310), and vaccinated pigs (*n* = 359), as described in the materials and methods section.

**Figure 3:**
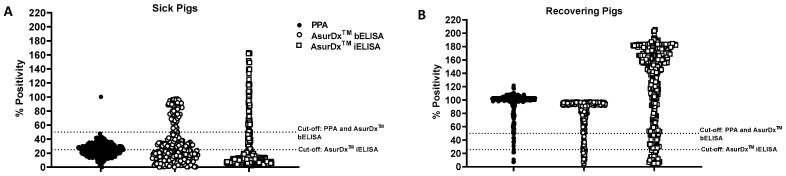
Performance of AsurDx^™^ ELISA kits using field samples. Samples obtained from various ASF-suspected farms in Vietnam were tested with AsurDx^™^ ELISA and PPA kits, and the percent positivity was calculated and graphically represented for the sick (**A**) and recovering pigs (**B**). The cut-off for each assay is shown with a broken line.

For samples from the sick pigs, PPA showed the lowest positivity rate (0.33%) compared to AsurDx^™^ bELISA (19.03%) and iELISA (26.45%), representing a significant discrepancy in the results (Figure 3A and Table 4). To resolve this, we tested all the discrepant samples using ASFV real-time PCR. Most (43 out of 46) of the AsurDx^™^ bELISA positive samples were positive for ASFV genomic material (with the remaining 3 pigs showing suspicious levels), confirming that the animals were indeed infected with ASFV and likely in the early stages (depicted by high ct values) of infection (Table S2A,B).

For the recovering pigs, the overall results yielded mixed outcomes, with the percent positivity for AsurDx^™^ bELISA and iELISA at 84.06% and 92.51%, respectively, compared to that of PPA, at 95.63% (Figure 3B and Table 4). Our attempt to address the discrepancies across the three kits using IPT was also challenging, with IPT data (for discrepant samples only) showing 20.00% positivity compared to PPA (100.00%), AsurDx^™^ bELISA (0.00%), and iELISA (50.00%) (Table S2C). It is worth noting that the recovering pig group comprised a mixture of ASF-positive and negative samples, with no way of determining the prior infection status of the pigs, hence the mixed results obtained with the assays.

**Figure 4:**
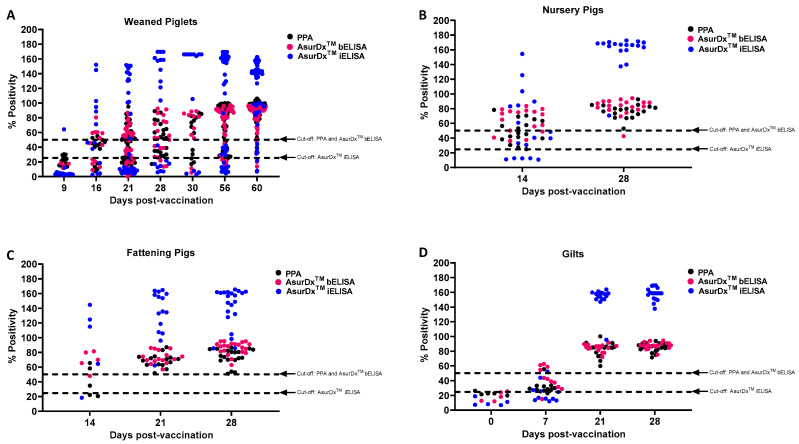
Performance of AsurDx^™^ ELISA kits using samples from vaccinated pigs. Samples obtained from vaccinated pigs in Vietnam were tested with AsurDx^™^ ELISA and PPA kits, and the percent positivity was calculated for the weaned piglets (**A**), nursery pigs (**B**), fattening pigs (**C**), and gilts (**D**). The cut-off for each assay is shown with a broken line.

Next, sera from four different age groups of pigs (weaned piglets, nursery pigs, fattening pigs and gilts) vaccinated with the live attenuated ASFV vaccine were tested. The samples from weaned piglets comprised sera collected on 9, 16, 21, 28, 30, 56, and 60 days post-vaccination (dpv). AsurDx^™^ iELISA detected anti-ASFV antibodies in one of the 13 sera collected as early as 9 dpv (Figure 4A and Table 5). The AsurDx^™^ iELISA detected anti-ASFV antibodies in 69.23% of the samples collected on 16 dpv, while the AsurDx^™^ bELISA identified anti-ASFV antibodies in 46.15% of the 16 dpv serum samples. In contrast, PPA could only detect anti-ASFV antibodies in 15.38% of the 16 dpv serum samples. However, by 60 DPV, all three assays were able to equally detect anti-ASF antibodies (95.45% for AsurDx^™^ bELISA, 96.59% for AsurDx^™^ iELISA, and 94.32% for PPA) (Figure 4A and Table 5).

In nursery pigs, AsurDx^™^ bELISA detected anti-ASFV antibodies in 76.47% of the sera collected on 14 dpv, while AsurDx^™^ iELISA detected anti-ASFV antibodies in 70.59% of the samples. The PPA detected anti-ASFV antibodies in 47.06% of the samples. By 28 dpv, all three assays were able to detect ASFV antibodies in almost all the samples (94.44–100.00%) (Figure 4B and Table 5).

Similarly, both AsurDx^™^ bELISA and AsurDx^™^ iELISA were able to detect anti-ASFV antibodies in 80% of the serum samples collected on 14 dpv from the vaccinated fattening pigs. In contrast, PPA was able to detect anti-ASFV antibodies only in 40% of the vaccinated fattening pigs (Figure 4C and Table 5). The three assays detected anti-ASFV antibodies (100%) in all the samples tested on 21 and 28 dpv.

In sera samples collected from vaccinated gilts, both AsurDx^™^ bELISA and AsurDx^™^ iELISA detected anti-ASFV antibodies in 36.36% of the serum samples collected on 7 dpv. In contrast, PPA detected anti-ASFV antibodies in only 9.09% of the serum samples. The three assays were able to detect anti-ASFV antibodies in 100% of the samples collected on 21 and 28 dpv (Figure 4D and Table 5).

### 3.6. Performance of AsurDx^™^ ELISA Kits with Meat Exudate from ASFV-Infected Pigs

Meat exudate (diaphragm) samples of different dpi from ASFV Malta’78-infected pigs were used to assess the performance of AsurDx^™^ bELISA and iELISA. Meat exudate samples are not recommended for testing using the PPA kit, a blocking ELISA assay similar to the AsurDx^™^ bELISA. With this in mind, developing an AsurDx^™^ iELISA kit was important to allow for the reliable testing of complex sample types, like meat exudate. Indeed, our data show that AsurDx^™^ bELISA and PPA produced false-positive results (high background) with meat exudate samples, compared to the results from the corresponding serum samples (Table 6). AsurDx™ iELISA was specific and sensitive with the same samples and gave comparable results with the corresponding serum samples, similar to ID Screen, known to be suitable for testing meat exudate samples (Table 6). Conclusively, our data indicate that while AsurDx™ iELISA performs very well and is suitable for testing meat exudate samples, AsurDx^™^ bELISA is not recommended due to the possibility of generating false-positive results as a result of a high signal background.

## 4. Discussion

Multiple studies have been conducted to evaluate recombinant ASFV antigens for the detection of antibodies to ASFV [16,32,33,34,35,36,37,38], and based on the studies, several commercial ELISA kits have been developed and licensed outside North America. This study describes the development and validation of two novel prototype commercial ELISA assays, AsurDx^™^ bELISA (ASFV p54) and AsurDx^™^ iELISA (ASFV p54 and p72), with relatively improved performance compared to the existing commercial ASF ELISA kits. The kits were extensively evaluated using a panel of well-characterized reference sera, sera collected from experimentally infected pigs, and field sera from ASF-free (Canada and USA) and ASF-endemic (Vietnam) countries.

In this study, we expressed and purified the highly conserved ASFV structural proteins p30, p54, and p72 using a baculovirus expression system. During the initial screening when a panel of known negative and positive serum samples was used, p54 showed the best sensitivity and specificity and the lowest cut-off threshold in the ROC analysis compared to p30, with the lowest level of performance. When the antigens were evaluated using a selected number of serial bleed serum samples from pigs experimentally infected with ASFV OURT88/3 and subsequently challenged with ASFV Malta’78, antibodies to p54 were detected as early as 8 dpi, two days before the antibodies to p72 were detected. In contrast, antibodies to p30 were not detected until 16 dpi (Figure 1B). Previous reports showed that p30-based iELISAs performed better than the p54-based version [34,36], which contradicts our study. The study by Giménez-Lirola et al. showed that anti-ASFV antibodies to *E. coli*-expressed ASFV p30 could be detected as early as 8 dpi by iELISA [34]. The exact reason for the discrepancy is not yet understood; however, it could be due to the differences between the two expression systems (*E. coli* vs. baculovirus) used. It is also possible that the His-tag attached to the N-terminus of the baculovirus-expressed p30 used in our study negatively affected antibody binding to the protein in iELISA. Due to the low performance level of baculovirus-expressed p30 in our initial screening assays, p54 and p72 were used for the development of iELISA and bELISA.

ASFV p72 is one of the first identified structural viral proteins responsible for the induction of antibodies; therefore, it is mostly used as an antibody detection target during ASFV infection studies [39]. Several ASFV p72-based indirect and blocking/competitive ELISA assays have been developed, with some of them available commercially [40,41,42]. Like p72, p54 is also a highly immunogenic structural protein of ASFV [43]. Using *E. coli*-expressed ASFV p54, Goa et al. and Tesfagaber et al. developed highly sensitive and specific blocking ELISAs for the detection of antibodies in ASFV-infected pigs [44,45]. A recent study used a baculovirus expression system to generate chimeric core-like particles with an ASFV p54 epitope, leading to the development of a highly sensitive and specific ASF blocking ELISA [46], further highlighting ASFV p54 as a target in ASF ELISA development.

The commercial PPA assay uses a mAb specific to ASFV p72 according to the manufacturer. The assay has previously shown 99% sensitivity compared to the OIE Reference Indirect ELISA [47]. However, in our study, when 61 pre-characterized reference sera saved from the annual proficiency panels from the EURL for ASF were used, the PPA assay showed 74.42% sensitivity compared to the AsurDx^™^ bELISA (97.67%) (Table 1). The reduced sensitivity of PPA is likely due to its inability to detect anti-ASFV antibodies during the early stages of infection. The positive serum panel used in this study contained sera collected from pigs between 7 and 206 dpi. The samples that tested negative by PPA were all under 21 dpi (Table 1), which is likely due to the low abundance of anti-p72 antibodies in those samples during the early phase of infection; this was further supported by the animal experiment data showing that the p54-based AsurDx^™^ bELISA detected anti-ASFV anti-bodies a few days earlier than the PPA (Table 3). A similar pattern was observed with the sera collected from sick pigs from outbreak farms in Vietnam (Table 4), where the AsurDx^™^ bELISA detected anti-ASFV antibodies before the PPA kit. All the pig serum samples that were positive on AsurDx^™^ bELISA from the outbreak farms were also positive for ASFV genomic DNA, confirming that those pigs were indeed infected with ASFV.

The AsurDx^™^ iELISA developed in this study was slightly less sensitive compared to the AsurDx^™^ bELISA; it failed to detect some of the weak anti-ASFV antibody-positive samples in the reference serum panel (Table 1). However, it showed 99.05–100% specificity when negative sera from the EURL, Canada, USA, and Vietnam were assessed (Table 1 and Table 2). In addition, its performance was comparable to that of the other assays when field sera collected from vaccinated, sick, and recovering pigs in Vietnam were assessed (Table 4 and Table 5). The main advantage of the AsurDx^™^ iELISA was apparent when meat exudate samples were tested. Both PPA and AsurDx^™^ bELISA kit assays gave false-positive results (Table 6) with meat exudate samples, whereas the AsurDx^™^ iELISA kit detected anti-ASF antibodies at levels comparable to the paired serum samples (Table 6). Similar results were obtained with the commercially available ID. Screen assay, which is recommended for testing meat exudates (Table 6).

In summary, we have developed and evaluated two novel highly sensitive and specific ELISA assays for the early detection of anti-ASFV antibodies in pigs infected with ASFV and those vaccinated with the live-attenuated ASFV vaccine. The ASFV p54-based AsurDx^™^ bELISA positively identified ASFV-infected and vaccinated pigs earlier than the commercially available PPA kit. The AsurDx^™^ bELISA could also be used to detect anti-ASFV antibodies in other pig species that are susceptible to ASFV, such as warthogs and bush pigs. The AsurDx^™^ iELISA is an equally sensitive and specific assay that can be used to detect anti-ASFV antibodies in serum and meat exudate samples from domestic and wild pigs. AsurDx^™^ bELISA and AsurDx^™^ iELISA are commercially available and can be used as alternatives to the other existing commercial ELISA assays for the serological detection of anti-ASFV antibodies during disease investigations, for post-outbreak surveillance, and to access vaccine uptake following live-attenuated ASFV vaccination.

## Figures and Tables

**Table 1:** Sensitivity and specificity of AsurDx^™^ ELISA kits using a panel of reference sera. The percentages of the positive, negative, and suspicious samples were calculated across AsurDx^™^ ELISA and PPA kits using a panel of 61 well-characterized reference sera to determine assay sensitivity—43 samples and specificity—18 samples. Pos, positive (green); Neg, negative (white); Sus, suspicious/Weak (orange).

Sensitivity of AsurDx^TM^ ELISA Kits									
						PPA	AsurDx^™^ bELISA		AsurDx^™^ iELISA
Sample ID	ASFV Isolate	Genotype	DPI	EURL Diagnostic Conclusion		Sample Blocking %	Inhibition %		% Positivity
S1	Est15/WB-Valga6	II	206	Positive		100.52	96.01		249.52
S2	NH/P68 + L60	I	50	Positive		96.28	95.33		248.49
S3	NH/P68	I	30	Positive		85.23	94.43		251.48
S4	E75 _CV1-4_ + E75	I	45	Positive		76.72	88.75		216.17
S5	Est15/WB-Valga6	II	206	Positive		100.46	95.87		234.56
S6	NH/P68	I	30	Positive		85.17	95.04		245.90
S7	NH/P68 + L60	I	50	Positive		97.28	95.52		265.37
S8	Ken05/Tk1	X	70	Positive		102.95	85.39		240.49
S9	646VR85	I	97	Positive		103.61	93.17		212.54
S10	NH/P68	I	72	Positive		101.79	94.88		241.47
S11	Est15/WBValga6	II	204	Positive		103.69	93.96		228.55
S12	646VR85	I	101	Positive		103.16	95.69		232.81
S13	NH/P68	I	45	Positive		100.45	95.65		225.56
S14	LIV17/WB/Rie14/Tukuma5	II	105	Positive		105.78	95.56		248.47
S15	LIV17/WB/Rie14/Tukuma5	II	105	Positive		105.73	95.26		249.25
S16	L60	I	7	Weak		19.12	60.69		9.78
S17	L60	I	7	Weak		23.15	62.26		10.60
S18	Est16/WB-VIRU8	II	76	Positive		105.28	90.91		233.50
S19	BEL18/WB-LUX1	II	10	Positive		55.13	82.72		60.47
S20	BEL18/WB-LUX1	II	10	Positive		49.75	80.08		58.96
S21	Est16/WB-VIRU8	II	76	Positive		104.03	88.90		238.22
S22	ET13/1505	XXIII	10	Positive		30.86	91.02		35.61
S23	ET13/1505	XXIII	10	Positive		29.65	90.46		41.66
S24	ET13/1505	XXIII	93	Positive		103.63	95.00		267.05
S25	ET13/1505	XXIII	23	Positive		56.08	73.91		57.79
S26	BEL18/WB-LUX1	II	14	Weak		−4.72	56.54		15.37
S27	LT1490	II	17	Weak		−31.63	45.95		14.70
S28	LT1490	II	17	Weak		−44.52	42.29		14.85
S29	LV17/WB-RIE1	II	101	Positive		103.03	95.61		238.40
S30	OURT88/3	I	97	Positive		103.43	93.99		256.78
S31	Est16/WB-VIRU8	II	76	Positive		102.75	89.42		236.15
S32	NH/P68	I	72	Positive		102.84	95.20		270.94
S33	NH/P68	I	52	Positive		103.11	94.25		254.14
S34	OURT88/3	I	97	Positive		102.18	95.33		269.22
S35	Pol16/DPOUT21	II	14	Weak		37.00	60.93		47.73
S36	LT1490	II	21	Weak		17.81	61.29		11.71
S37	Pol16/DPOUT21	II	20	Positive		76.14	53.55		136.56
S38	Arm07	II	7	Weak		9.04	34.86		7.46
S39	NH/P68	I	36	Positive		96.37	95.74		214.77
S40	ET13/1505	XXIII	93	Positive		100.92	95.59		197.92
S41	NH/P68	I	117	Positive		101.47	97.29		216.07
S42	Est15/WB-Valga6	II	204	Positive		101.78	97.20		191.81
S43	Est15/WBTartu14	II	206	Positive		101.28	96.83		187.16
				Sensitivity		32 out of 43	42 out of 43		36 out of 43
						74.42%	97.67%		83.72%
**Specificity of AsurDx^TM^ ELISA Kits**									
					**PPA**	**AsurDx^™^ bELISA**		**AsurDx^™^ iELISA**	
**Sample** **ID**	**Sample Name**	**EURL Diagnostic Conclusion**			**Sample** **Blocking %**	**Inhibition %**		**% Positivity**	
S1	Naive Pig	Negative			27.76	13.79		9.13	
S2	Naive Pig	Negative			6.11	13.86		9.43	
S3	Naive Pig	Negative			14.53	20.13		7.99	
S4	Naive Pig	Negative			7.24	26.74		10.21	
S5	Naive Pig	Negative			5.31	4.45		11.10	
S6	Naive Pig	Negative			−5.42	7.23		8.47	
S7	Naive Pig	Negative			3.95	10.49		13.15	
S8	Naive Pig	Negative			19.35	7.10		8.88	
S9	Naive Pig	Negative			13.70	14.84		9.10	
S10	Naive Pig	Negative			14.55	13.69		9.19	
S11	Naive Pig	Negative			9.00	14.08		10.05	
S12	Naive Pig	Negative			−3.32	13.15		10.02	
S13	Naive Pig	Negative			−20.84	21.34		9.42	
S14	Naive Pig	Negative			11.16	4.75		4.68	
S15	Naive Pig	Negative			5.24	−0.39		4.08	
S16	Naive Pig	Negative			25.22	0.20		5.04	
S17	Naive Pig	Negative			21.83	1.67		4.00	
S18	Naive Pig	Negative			−1.12	23.98		6.71	
		Specificity			18 out of 18	18 out of 18		18 out of 18	
					100.00%	100.00%		100.00%	

**Table 2:** Performance of AsurDx^™^ ELISA kits with ASF-negative samples from North America and Vietnam. The performance of the AsurDx^™^ ELISA kits was assessed using ASF-negative samples from North America and Vietnam, and the percentages of the positive, negative, and suspicious samples were calculated across AsurDx^™^ ELISA and PPA kits. Pos, positive; Neg, negative; Sus, suspicious.

Performance of AsurDx^™^ bELISA and iELISA with ASF-Negative Samples																			
	Combined (Domestic Pigs and Wild Boars): 2792				Healthy Domestic Pigs (Canada): 2406					Wild Boars (USA): 200					Wild Boars (Canada): 186				
	Pos.	Neg.		Sus.	Pos.		Neg.		Sus.	Pos.		Neg.		Sus.	Pos.		Neg.		Sus.
**PPA**	0 (0.00%)	2790 (99.93%)		2 (0.07%)	0 (0.00%)		2404 (99.92%)		2 (0.08%)	0 (0.00%)		200 (100%)		0 (0.00%)	0 (0.00%)		186 (100%)		0 (0.00%)
**AsurDx™ bELISA**	11 (0.39%)	2773 (99.32%)		8 (0.29%)	5 (0.21%)		2398 (99.67%)		3 (0.12%)	2 (1.00%)		195 (97.50%)		3 (1.50%)	4 (2.15%)		180 (96.77%)		2 (1.08%)
**AsurDx™ iELISA**	15 (0.54%)	2774 (99.36%)		3 (0.10%)	9 (0.37%)		2394 (99.50%)		3 (0.12%)	2 (1.00%)		198 (99.00%)		0 (0.00%)	4 (2.15%)		182 (97.85%)		0 (0.00%)
**Healthy Domestic Pigs (Vietnam)**																			
**PPA: 210**						**AsurDx^™^ bELISA: 210**							**AsurDx^™^ iELISA: 210**						
Pos	Neg.		Sus.			Pos.		Neg.			Sus.		Pos.			Neg.		Sus.	
0 (0.00%)	209 (99.52%)		1 (0.48%)			1 (0.48%)		207 (98.57%)			2 (0.95%)		2 (0.95%)			208 (99.05%)		0 (0.00%)	

**Table 3:** Tabular representation of the performance of AsurDx^™^ ELISA, PPA, and ID. Screen kits. Pigs were experimentally infected with ASFV Estonia 2014, ASFV Malta’78, ASFV Georgia 2007/1, ASFV OURT88/3, and ASF-GUS-Vietnam, and the serial bleed serum samples were collected at the indicated time points. The assay performance was assessed and compared between AsurDx^™^ ELISA, PPA, and ID. Screen kits. Negative (white); suspicious (orange); positive (green). NT, not tested.

ASFV Estonia 2014				
	PPA	AsurDx^™^ bELISA	AsurDx^™^ iELISA	ID. Screen
Sample Name	Sample Blocking %	Inhibition %	% Positivity	S/P %
Pig 3, DPI 5	12.97	−8.82	2.20	0.34
Pig 13, DPI 5	16.24	−4.26	12.45	0.17
Pig 29, DPI 5	15.81	−1.02	4.45	0.81
Pig 36, DPI 5	22.06	−5.44	2.77	0.35
Pig 10, DPI 7	33.46	52.54	15.07	2.34
Pig 16, DPI 7	14.58	35.61	4.70	1.03
Pig 28, DPI 7	25.83	36.66	6.57	0.45
Pig 31, DPI 7	18.78	39.70	11.74	0.96
Pig 17, DPI 8	21.68	60.05	10.02	1.11
Pig 19, DPI 8	56.53	73.88	36.19	4.40
Pig 23, DPI 8	38.92	71.83	78.54	3.34
Pig 25, DPI 8	40.83	54.40	29.08	1.42
Pig 8, DPI 9	45.10	75.93	51.67	6.99
Pig 21, DPI 9	44.88	92.42	100.05	27.18
Pig 24, DPI 9	36.74	75.66	76.58	18.60
Pig 7, DPI 10	37.50	81.43	68.56	4.29
Pig 32, DPI 10	84.23	74.69	139.51	48.58
Pig 4, DPI 11	83.03	94.37	127.94	58.43
Pig 12, DPI 11	46.01	67.41	115.43	30.74
**ASFV Malta’78**				
	**PPA**	**AsurDx^™^** **bELISA**	**AsurDx^™^ iELISA**	**ID. Screen**
**Sample Name**	**Sample** **Blocking %**	**Inhibition %**	**%Positivity**	**S/P %**
Pig 72, DPI 5	22.81	3.91	2.36	−0.04
Pig 71, DPI 5	18.82	3.99	3.20	0.47
Pig 53, DPI5	19.83	2.71	2.29	0.23
Pig 46, DPI 5	16.81	9.90	2.54	0.21
Pig 80, DPI 6	16.17	7.86	1.87	0.53
Pig 70, DPI 7	27.34	50.84	1.94	−0.10
Pig 68, DPI 7	26.93	47.31	1.89	0.78
Pig 54, DPI 7	28.43	36.67	1.83	0.75
Pig 48, DPI 7	31.92	48.79	1.80	0.65
Pig 79, DPI 9	67.37	76.24	2.02	25.38
Pig 58, DPI 9	59.42	70.88	2.25	15.81
Pig 50, DPI 9	80.83	80.94	2.12	23.87
Pig 75, DPI 10	67.29	75.85	35.24	22.80
Pig 67, DPI 11	70.72	88.44	94.32	33.17
Pig 62, DPI11	38.99	79.53	78.50	17.08
Pig 47, DPI 12	63.81	69.34	96.44	36.20
Pig 49, DPI 12	71.82	67.56	25.56	54.15
Pig 63, DPI 13	55.34	78.11	102.63	66.20
Pig 45, DPI 13	55.59	74.17	140.19	59.91
Pig 57, DPI 16	61.83	69.30	175.81	70.81
Pig 51, DPI 18	63.47	84.08	176.66	70.12
Pig 56, DPI 18	77.43	74.38	144.64	86.03
Pig 66, DPI 18	76.40	84.90	182.03	81.45
**ASFV Georgia 2007/1**				
	**PPA**	**AsurDx^™^ bELISA**	**AsurDx^™^ iELISA**	**ID. Screen**
**Sample**	**Sample** **Blocking %**	**Inhibition %**	**% Positivity**	**S/P %**
Pig 118, DPI 6	15.216	−17.011	5.54	0.63
Pig 125, DPI 6	18.310	−9.576	3.40	0.38
Pig 108, DPI 7	22.765	−12.003	2.13	0.39
Pig 110, DPI 7	21.632	−9.454	5.08	0.46
Pig 118, DPI 8	12.243	−9.365	5.42	0.52
Pig 125, DPI 8	16.850	−10.457	3.56	0.52
Pig 108, DPI 9	31.211	−3.546	2.80	0.33
Pig 110, DPI 9	21.465	−7.894	6.24	0.81
Pig 118, DPI 10	8.860	−13.530	22.45	0.68
Pig 125, DPI 10	12.996	−13.155	3.15	0.44
Pig 108, DPI 11	32.009	24.432	201.94	0.97
Pig 110, DPI 11	12.046	5.987	5.63	0.53
Pig 118, DPI 12	9.636	67.412	8.37	0.82
Pig 125, DPI 12	10.525	−6.372	3.92	0.37
Pig 108, DPI 13	49.768	66.100	108.72	16.23
Pig 110, DPI 13	24.612	79.597	39.99	4.28
Pig 118, DPI 14	37.224	87.262	42.56	7.49
Pig 125, DPI 14	27.007	50.682	10.77	2.76
Pig 110, DPI 16	71.275	80.867	161.99	26.94
Pig 118, DPI 16	78.801	87.182	101.99	40.84
Pig 108, DPI 17	75.798	64.001	102.97	77.05
Pig 125, DPI 18	81.842	76.842	63.63	51.70
**ASFV OURT88/3**				
	**PPA**	**AsurDx^™^ bELISA**	**AsurDx^™^ iELISA**	**ID. Screen**
**Sample Name**	**Sample Blocking %**	**Inhibition %**	**% Positivity**	**S/P %**
Pig 1, DPI 6	10.17	3.12	4.15	0.60
Pig 2, DPI 6	5.30	2.59	5.47	0.51
Pig 3, DPI 6	−25.21	3.08	3.37	NT
Pig 4, DPI 6	13.20	1.52	6.98	1.08
Pig 1, DPI 8	38.44	48.42	28.59	25.35
Pig 2, DPI 8	6.48	21.90	10.53	2.40
Pig 3, DPI 8	23.90	61.10	27.89	14.50
Pig 4, DPI 8	24.15	45.37	29.19	13.12
Pig 1, DPI 10	72.41	66.30	113.57	65.89
Pig 2, DPI 10	98.45	68.46	166.57	37.52
Pig 3, DPI 10	38.02	70.64	107.86	41.11
Pig 4, DPI 10	39.97	52.22	99.80	37.41
Pig 1, DPI 12	64.74	70.39	164.96	75.23
Pig 2, DPI 12	98.07	77.23	165.49	63.69
Pig 3, DPI 12	42.18	58.05	130.73	49.85
Pig 4, DPI 12	47.03	58.66	121.74	59.94
Pig 4, DPI 14	54.70	51.01	119.41	67.31
Pig 3, DPI 14	49.97	54.03	152.11	63.60
Pig 2, DPI 14	99.14	76.93	167.09	77.58
Pig 1, DPI 14	74.97	68.37	158.15	87.41
Pig 1, DPI 16	70.31	67.26	163.60	82.24
Pig 2, DPI 16	95.58	75.18	144.70	87.98
Pig 3, DPI 16	61.74	51.52	162.51	52.30
Pig 4, DPI 16	50.71	51.41	132.69	70.19
**ASF-GUS-Vietnam**				
	**PPA**	**AsurDx^™^ bELISA**	**AsurDx^™^ iELISA**	**ID. Screen**
**Sample Name**	**Sample Blocking %**	**Inhibition %**	**% Positivity**	**S/P %**
P2, DPI 6	23.18	4.78	1.98	1.18
P3, DPI 6	24.79	11	3.2	0.6
P4, DPI 6	21.77	9.73	2.12	4.32
P5, DPI 6	24.21	−12.25	2.83	2.64
P6, DPI 6	19.77	26.63	1.58	1.07
P2, DPI 9	40.25	47.65	9.32	32.99
P3, DPI 9	31.64	67.37	12.71	7.82
P4, DPI 9	42.05	66.42	21.24	35.47
P5, DPI 9	18.4	0.84	2.89	2.17
P6, DPI 9	23.34	63.63	18.69	24.23
P2, DPI 12	55.67	69.53	1.62	52.17
P3, DPI 12	54.06	80.25	107.28	60.32
P4, DPI 12	50.9	73.3	78.29	70.89
P5, DPI 12	25	5.53	2.66	4.48
P6, DPI 12	45.27	75.15	114.87	67.12
P2, DPI 14	72.71	71.74	107.39	79.37
P3, DPI 14	79.6	81.03	132.92	81.79
P4, DPI 14	65.14	72.84	118.67	82.58
P5, DPI 14	37.16	1.52	3.44	14.27
P6, DPI 14	58.8	73.06	142.9	78.33
P2, DPI 18	90	68.23	132.4	88.23
P3, DPI 18	93.56	77.81	147.53	93.26
P4, DPI 18	66.3	79.38	145.41	79.27
P5, DPI 18	38.02	12.59	16.62	23.51
P6, DPI 18	60.32	74.7	143.77	91.7
P2, DPI 21	94.26	67.9	143.25	95.08
P3, DPI 21	97.79	95.7	148.35	94.04
P4, DPI 21	76.12	80.85	152.65	86.49
P5, DPI 21	45.98	18.36	19.99	71.19
P6, DPI 21	65.37	80.79	146.92	83.69
P2, DPI 27	95.26	86.41	144.77	81.18
P3, DPI 27	98.53	97.31	150.06	92.92
P4, DPI 27	81.74	84.48	152.24	79.66
P5, DPI 27	78.51	74.13	139.43	96.61
P6, DPI 27	75.66	93.01	146.45	92.04

**Table 4:** Tabular representation of AsurDx^™^ ELISA kits’ performance using field samples. The percentages of positive, negative, and suspicious results were calculated for samples from sick and recovering pigs following testing with AsurDx^™^ ELISA and PPA kits.

Sick Pigs								
PPA: 310			AsurDx^™^ bELISA: 310			AsurDx^™^ iELISA: 310		
Pos.	Neg.	Sus.	Pos.	Neg.	Sus.	Pos.	Neg.	Sus.
1 (0.33%)	300 (96.77%)	9 (2.90%)	59 (19.03%)	241 (77.74%)	10 (3.23%)	82 (26.45%)	219 (70.65%)	9 (2.90%)
**Recovering Pigs**								
**PPA: 412**			**AsurDx^™^ bELISA: 414**			**AsurDx^™^ iELISA: 414**		
**Pos.**	**Neg.**	**Sus.**	**Pos.**	**Neg.**	**Sus.**	**Pos.**	**Neg.**	**Sus.**
394 (95.63%)	14 (3.40%)	4 (0.97%)	348 (84.06%)	60 (14.49%)	6 (1.45%)	383 (92.51%)	28 (6.76%)	3 (0.73%)

**Table 5:** Tabular representation of the AsurDx^™^ ELISA kits’ performance using samples from vaccinated pigs. The percentages of positive, negative, and suspicious results were calculated for samples obtained from weaned piglets, nursery pigs, fattening pigs, and gilts following testing with AsurDx^™^ ELISA and PPA kits.

Weaned Piglets		
9 DPV		
PPA: 13	AsurDx^™^ bELISA: 13	AsurDx^™^ iELISA: 13
0 (0.00%)	0 (0.00%)	1 (7.69%)
16 DPV		
PPA: 13	AsurDx^™^ bELISA: 13	AsurDx^™^ iELISA: 13
2 (15.38%)	6 (46.15%)	9 (69.23%)
21 DPV		
PPA: 38	AsurDx^™^ bELISA: 39	AsurDx^™^ iELISA: 38
17 (44.74%)	18 (46.15%)	17 (44.74%)
28 DPV		
PPA: 20	AsurDx^™^ bELISA: 20	AsurDx^™^ iELISA: 20
12 (60.00%)	11 (55.00%)	12 (60.00%)
30 DPV		
PPA: 13	AsurDx^™^ bELISA: 13	AsurDx^™^ iELISA: 13
8 (61.54%)	9 (69.23%)	9 (69.23%)
56 DPV		
PPA: 47	AsurDx^TM^ bELISA: 47	AsurDx^™^ iELISA: 47
34 (72.34%)	31 (65.96%)	35 (74.47%)
60 DPV		
PPA: 88	AsurDx^™^ bELISA: 88	AsurDx^™^ iELISA: 88
83 (94.32%)	84 (95.45%)	85 (96.59%)
**Nursery Pigs**		
14 DPV		
PPA: 17	AsurDx^™^ bELISA: 17	AsurDx^™^ iELISA: 17
8 (47.06%)	13 (76.47%)	12 (70.59%)
28 DPV		
PPA: 18	AsurDx^™^ bELISA: 18	AsurDx^™^ iELISA: 18
18(100.00%)	17 (94.44%)	18 (100.00%)
**Fattening Pigs**		
14 DPV		
PPA: 5	AsurDx^™^ bELISA: 5	AsurDx^™^ iELISA: 5
2 (40.00%)	4 (80.00%)	4 (80.00%)
21 DPV		
PPA: 15	AsurDx^™^ bELISA: 15	AsurDx^™^ iELISA: 15
15 (100.00%)	15 (100.00%)	15 (100.00%)
28 DPV		
PPA: 19	AsurDx^™^ bELISA: 21	AsurDx^™^ iELISA: 21
19 (100.00%)	21 (100.00%)	21 (100.00%)
**Gilts**		
0 DPV		
PPA: 5	AsurDx^™^ bELISA: 5	AsurDx^™^ iELISA: 5
0 (0.00%)	0 (0.00%)	0 (0.00%)
7 DPV		
PPA: 11	AsurDx^™^ bELISA: 11	AsurDx^™^ iELISA: 11
1 (9.09%)	4 (36.36%)	4 (36.36%)
21 DPV		
PPA: 15	AsurDx^™^ bELISA: 16	AsurDx^™^ iELISA: 16
15 (100.00%)	16 (100.00%)	16 (100.00%)
28 DPV		
PPA: 18	AsurDx^™^ bELISA: 18	AsurDx^™^ iELISA: 18
18 (100.00%)	18 (100.00%)	18 (100.00%)

**Table 6:** Assessment of AsurDx^™^ ELISA kits’ performance with meat (diaphragm) exudate. Pigs were experimentally infected with ASFV Malta’78, and serial bleed serum samples were collected at the indicated time points. The assay performance was assessed and compared between PPA, AsurDx^™^ ELISA, and ID. Screen kits. Negative (white); suspicious (orange); positive (green).

	PPA (Sample Blocking %)		AsurDx^™^ bELISA (Inhibition %)		AsurDx^™^ iELISA (% Positivity)		ID. Screen (S/P %)	
Sample Name	Serum	Diaphragm	Serum	Diaphragm	Serum	Diaphragm	Serum	Diaphragm
Pig 72, DPI 5	22.81	62.46	3.91	67.77	2.36	2.21	−0.04	0.39
Pig 71, DPI 5	18.82	48.31	3.99	64.97	3.2	2.21	0.47	1.33
Pig 53, DPI5	19.83	50.56	2.71	62.07	2.29	2.2	0.23	0.29
Pig 46, DPI 5	16.81	53.78	9.9	66.15	2.54	2.17	0.21	0.25
Pig 80, DPI 6	16.17	53.67	7.86	50.51	1.87	2.24	0.53	1.04
Pig 70, DPI 7	27.34	54.94	50.84	62	1.94	2.11	−0.1	1.73
Pig 68, DPI 7	26.93	47.87	47.31	53.97	1.89	2.13	0.78	1.4
Pig 54, DPI 7	28.43	49.02	36.67	53.61	1.83	2.18	0.75	0.89
Pig 48, DPI 7	31.92	58.31	48.79	60.22	1.8	2.14	0.65	1.59
Pig 79, DPI 9	67.37	65.24	76.24	73.23	2.02	9.15	25.38	18.26
Pig 58, DPI 9	59.42	76.19	70.88	59.62	2.25	2.41	15.81	16.17
Pig 50, DPI 9	80.83	68.41	80.94	67.91	2.12	2.23	23.87	17.36
Pig 75, DPI 10	67.29	80.26	75.85	60.27	35.24	25.42	22.8	30.78
Pig 67, DPI 11	70.72	52.72	88.44	77.38	94.32	41.76	33.17	26.73
Pig 62, DPI11	38.99	73.72	79.53	66.09	78.5	17.76	17.08	14.96
Pig 47, DPI 12	63.81	62.06	69.34	49.25	96.44	19.23	36.2	45.48
Pig 49, DPI 12	71.82	72.44	67.56	66.83	25.56	94.06	54.15	37.77
Pig 63, DPI 13	55.34	65.24	78.11	71.64	102.63	141.15	66.2	73.01
Pig 45, DPI 13	55.59	62.82	74.17	62.82	140.19	147.99	59.91	60.44
Pig 57, DPI 16	61.83	81.19	69.3	54.19	175.81	207.89	70.81	77.22
Pig 51, DPI 18	63.47	70.03	84.08	76.11	176.66	189.49	70.12	67.58
Pig 56, DPI 18	77.43	79.6	74.38	54.63	144.64	174.52	86.03	90.9
Pig 66, DPI 18	76.4	79.53	84.9	74.15	182.03	112.96	81.45	80.69

## Data Availability

The authors will provide the data related to this manuscript upon request.

## Supplementary Materials

The following supporting information can be downloaded at: https://www.mdpi.com/article/10.3390/pathogens13110981/s1, Figure S1: Coomassie-stained gel (A) and Western blot (B) of recombinant ASFV p30, p54, and p72; Table S1: Diagnostic performance of ASFV iELISA under comparison; Figure S2: Performance of AsurDx^™^ ELISA kits using different strains and genotypes of ASFV; Table S2: Analysis of discordant samples from sick and recovering pigs.
