# Supplementary material for: Development and Validation of an Indirect and Blocking ELISA for the Serological Diagnosis of African Swine Fever
Source: Pathogens. 2024 Nov 8;13(11):981. doi: 10.3390/pathogens13110981 (PMC11597605; doi:10.3390/pathogens13110981)
Supplement: Supplementary file 1 [file pathogens-13-00981-s001.zip › pathogens-3279179-supplementary.pdf]

Article

Supplementary data:

Development and validation of an indirect and a blocking ELISA for serological diagnosis of African swine fever

Chukwunonso Onyilagha[1#*], Kaye Quizon[2#], Dmytro Zhmendak[1], Ian El Kanoa[1], Thang Truong[2], Thanuja Ambagala[1], Alfonso Clavijo[3], Van Phan Le[4], Shawn Babiuk[1,5], Aruna Ambagala[1,6*]

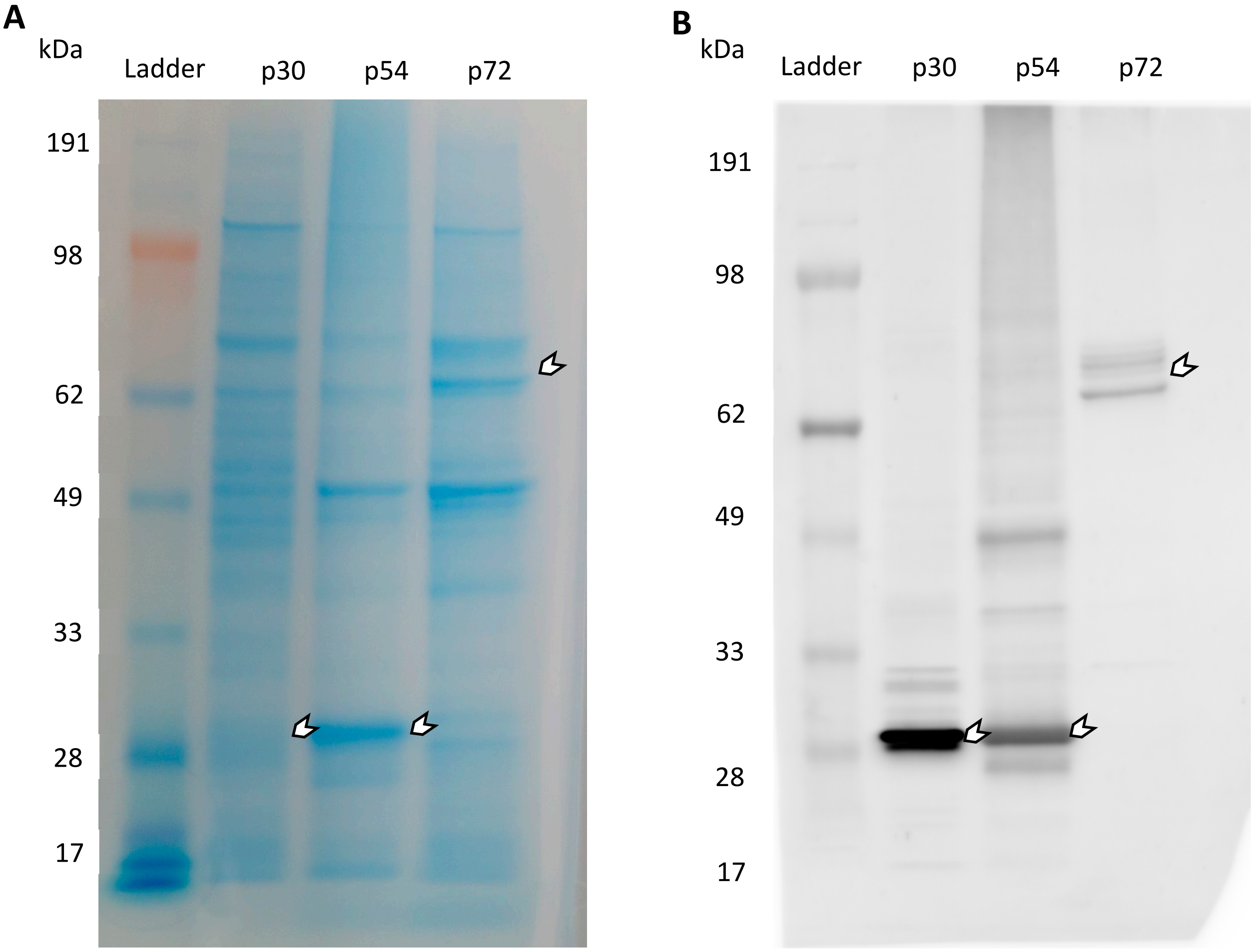

Figure S1. Coomassie-stained gel (A) and Western blot (B) of recombinant ASFV p30, p54, and p72. Proteins were expressed in Trichoplusia ni (Tni)cells grown in ESF 921 media using a baculovirus expression vector. The mass standard is SeeBlue2. Expected sizes: p30 = 23kDa; p54 = 20kDa; p72 = 73kDa. Arrows indicate the proteins of interest.

Table S1. Diagnostic performance of ASFV iELISA under comparison. AUC = Area under the curve; 95%CI = 95% confidence interval. a, sensitivity is defined as the true positive rate; b, specificity here is given as the true negative rate.

ELISA	Sample population		AUC(95%CI)	Cut-off Threshold	[a]Sensitivity	[b]Specificity
	Negative Sera (n)	Positive Sera (n)				
p30 indirect	353	85	0.843 (0.761, 0.925)	15.5%	83.5%	83.3%
p54 indirect	458	147	0.942 (0.898, 0.986)	10.5%	88.4%	94.8%
p72 indirect	352	85	0.724 (0.646, 0.802)	36.5%	51.8%	83.0%

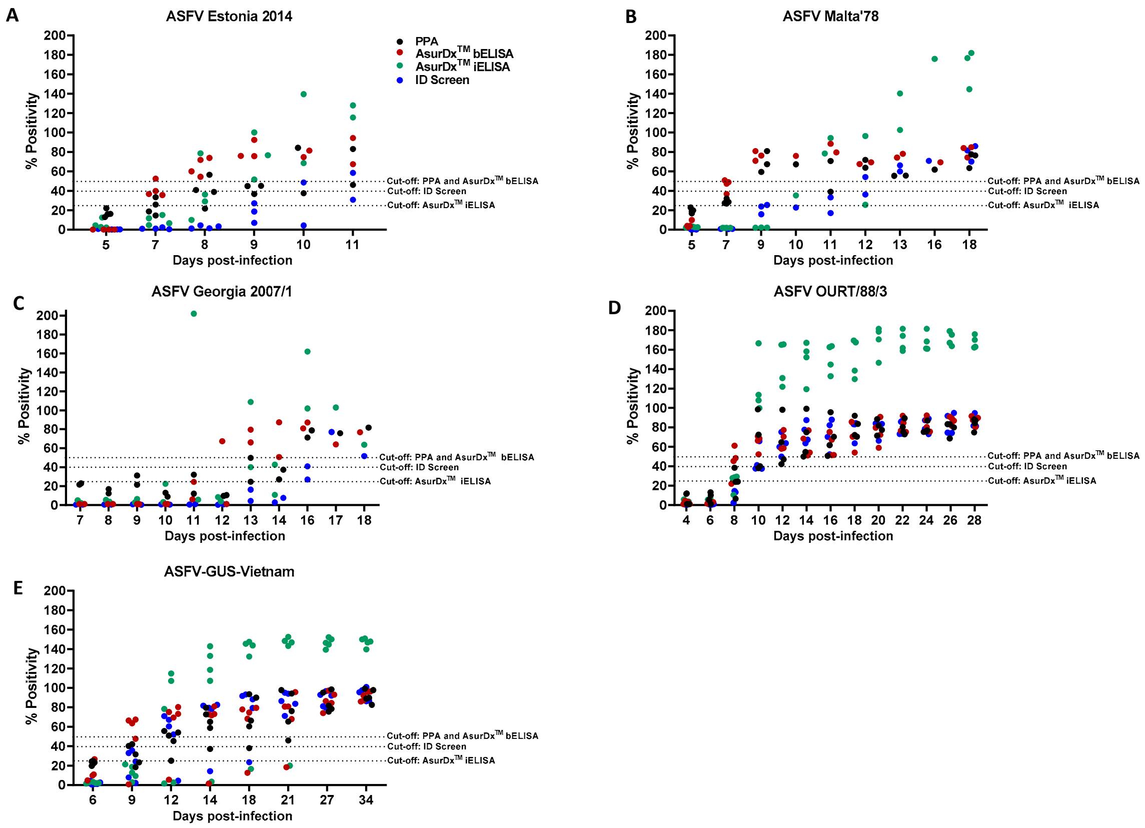

Figure S2: *Performance of AsurDx™ ELISA kits using different strains and genotypes of ASFV.* Groups of pigs were experimentally infected with ASFV Estonia 2014 (A), ASFV Malta'78 (B), ASFV Georgia 2007/1 (C), ASFV OURT88/3 (D), and ASFV-GUS-Vietnam (E) and the serial bleed serum samples were collected at indicated time points and tested with AsurDx™ ELISA, PPA, and ID. Screen kits to determine the assay performance. The cut-off for each assay is shown with a broken line.

Table S2. Analysis of discordant samples from sick and recovering pigs. Forty-six discordant samples from sick pigs (AsurDx™ ELISA kits and PPA) were tested by ASFV real-time PCR, and all the samples had Ct values between 19.92 and 37.44 (A & B). Table C shows the IPT results for 10 discordant samples from the recovering pigs.

A

Serial number	Sample ID (#)	ASFV Real-time PCR value
1	66	22.30
2	74	36.34
3	80	26.10
4	82	25.14
5	83	26.78
6	84	19.92
7	85	27.87
8	86	24.95
9	96	22.35
10	100	25.38
11	101	25.88
12	102	23.05
13	106	21.10
14	141	37.44
15	142	23.66
16	143	23.78
17	157	29.89
18	158	33.80
19	163	34.63
20	164	31.72
21	165	34.00
22	174	32.03
23	177	31.82
24	178	29.46
25	179	32.94
26	180	29.30
27	181	37.31
28	207	25.80
29	208	23.63
30	209	24.44
31	210	30.55
32	213	32.18
33	214	27.87
34	215	26.24
35	216	28.04
36	217	33.48
37	218	29.82
38	220	27.34
39	221	22.67
40	223	31.34
41	224	28.65
42	226	27.45
43	227	29.03
44	228	28.61
45	229	28.08
46	239	32.15

B

Discordant Samples from Sick Pigs											
**IPT: 46**			**PPA: 46**			**AsurDx™ bELISA: 46**			**AsurDx™ iELISA: 46**		
Pos.	Neg.	Sus.	Pos.	Neg.	Sus.	Pos.	Neg.	Sus.	Pos.	Neg.	Sus.
0 (0.00%)	44 (95.65%)	2 (4.35%)	0 (0.00%)	40 (86.96%)	6 (13.04%)	39 (84.78%)	2 (4.35%)	5 (10.87%)	40 (86.96%)	3 (6.52%)	3 (6.52%)

C

Discordant Samples from Recovering Pigs											
**IPT: 10**			**PPA: 10**			**AsurDx™ bELISA: 10**			**AsurDx™ iELISA: 10**		
Pos.	Neg.	Sus.	Pos.	Neg.	Sus.	Pos.	Neg.	Sus.	Pos.	Neg.	Sus.
2 (20.00%)	5 (50.00%)	3 (30.00%)	10 (100.00%)	0 (0.00%)	0 (0.00%)	0 (0.00%)	9 (90.00%)	1 (10.00%)	5 (50.00%)	4 (40.00%)	1 10.00%)
